# Application of FLAIR Vascular Hyperintensity-DWI Mismatch in Ischemic Stroke Depending on Semi-Quantitative DWI-Alberta Stroke Program Early CT Score

**DOI:** 10.3389/fneur.2019.00994

**Published:** 2019-09-26

**Authors:** Lei Song, Cui Lyu, Guiquan Shen, Tingting Guo, Jiangtao Wang, Wanbi Wang, Xiaoming Qiu, Alexander Lerner, Max Wintermark, Bo Gao

**Affiliations:** ^1^Department of Radiology, Affiliated Hospital of Guizhou Medical University, Guiyang, China; ^2^Department of Radiology, Xiangyang Central Hospital, Affiliated Hospital of Hubei University of Arts and Science, Xiangyang, China; ^3^Healthcare Examination Center, Affiliated Hospital of Guizhou Medical University, Guiyang, China; ^4^Department of Radiology, Xiangyang No. 1 People's Hospital, Hubei University of Medicine, Xiangyang, China; ^5^Department of Radiology, Huangshi Central Hospital, Affiliated Hospital of Hubei Polytechnic University, Edong Healthcare Group, Huangshi, China; ^6^Division of Neuroradiology, Department of Radiology, Keck School of Medicine University of Southern California, Los Angeles, CA, United States; ^7^Neuroradiology Section, Department of Radiology, Stanford University School of Medicine, Stanford, CA, United States

**Keywords:** cerebral infarction, magnetic resonance imaging, ASPECTS, fluid-attenuated inversion recovery vascular hyperintensity, stroke

## Abstract

**Objective:** Diffusion-weighted imaging (DWI)-Alberta Stroke Program Early CT Score (ASPECTS) is a simple, widely used method to estimate the size of the infarct. Our aim is to determine whether there is a relationship between DWI-ASPECTS and fluid-attenuated inversion recovery (FLAIR) vascular hyperintensity (FVH)-DWI mismatch and to better quantify FVH-DWI mismatch to assess the prognosis of cerebral infarction.

**Materials and Methods:** A retrospective analysis of 109 patients with MCA stenosis or occlusion with cerebral infarction was performed by dividing this cohort into FVH-DWI match group and FVH-DWI mismatch group based on FVH and DWI results. The clinical and imaging data of these two groups of patients were reviewed and analyzed to identify associations between FVH-DWI mismatch and prognosis of patients for preservation of neurological function. Correlation between DWI-ASPECTS and FVH-DWI mismatch was also performed.

**Results:** FVH-DWI mismatch was present in 66/109 (60.55%) patients, and FVH-DWI match was present in 43/109 (39.45%). Patients with FVH-DWI mismatch had higher DWI-ASPECTS (7.0 vs. 4.0, *P* < 0.001) and lower mRS at 3 months (3.0 vs. 4.0, *P* < 0.001) than patients without FVH-DWI mismatch. Multiple regression analysis suggested that DWI-ASPECTS (OR = 4.7, 95% CI = 2.5–9.2, *P* < 0.001) remained significantly associated with FVH-DWI mismatch. Two threshold points for DWI-ASPECTS of 3 and 8 can be used to distinguish whether there is a mismatch in FVH-DWI by smooth curve fitting.

**Conclusions:** The DWI-ASPECTS score was an independent predictor of FVH-DWI mismatch. At DWI-ASPECTS ≤ 3, the FVH-DWI mismatch offers no prognostic value; whereas, at DWI-ASPECTS ≥ 8, the FVH-DWI mismatch had the highest prognostic value. DWI-ASPECTS can roughly determine whether there is a FVH-DWI mismatch in order to select optimal clinical treatment and accurately assess prognosis.

## Introduction

Accurate assessment of the prognosis of ischemic stroke can help in selection of optimal treatment and may improve patient survival rate and reduce the rate of disability (1–3). Greater collateral circulation can reduce the infarct size, improve the patient's clinical prognosis, and further reduce the risk of recurrence (4). The latest DEFUSE 3 trial showed that large vessel occlusion thrombectomy in patients within 6–16 h after the onset of stroke resulted in lower disability and higher functional independence at 3 months, and this study have revealed that collateral circulation plays an important role in predicting outcomes (1). Multiple previous studies have explored the use of non-invasive angiography for assessment of collateral circulation after ICA occlusion. Fluid-attenuated inversion recovery vascular hyperintensity (FVH) has also been widely studied for such assessment. The current investigations of the relationship between FVH and DWI have suggest that FVH-DWI mismatch rather than FVH-DWI match can better predict prognosis (5–8). Several previous studies suggested that FVH-DWI mismatch can help assess the clinical neurological outcome, ischemic penumbra and thrombolytic therapy in patients with acute cerebral infarction (5, 7, 8). FVH-DWI mismatch has high sensitivity to PWI-DWI mismatch and can therefore be used to rapidly identify acute ischemic stroke patients with proximal vascular occlusion and reperfusion therapy (7). However, there are no unified FVH quantitative assessment methods to discriminate the relationship between FVH and DWI (7–9).

The Alberta Stroke Program Early CT Score (ASPECTS) is simple and semi-quantitative scoring systems that evaluate early ischemic changes in the middle cerebral artery territory (10). However, DWI-ASPECTS has great advantages compared to ASPECTS. This MRI based scoring system is more sensitive and consistent in detecting ischemic changes than CT and it can measure the volume of the lesions quickly and reliably. ASPECTS was used to approximately estimate the extent of hypoperfusion and the perfusion-weighted imaging (PWI)-DWI mismatch (11, 12). Above all, these prior investigations have not studied the relationship between FVH-DWI mismatch and DWI-ASPECTS. The aim of this study is to evaluate the association between FVH-DWI mismatch and DWI-ASPECTS as well as semi-quantitatively distinguish FVH-DWI mismatch and FVH-DWI match by means of DWI-ASPECTS. The hypothesis is that the association between FVH-DWI mismatch and DWI-ASPECTS would be better in acute ischemic stroke patients.

## Materials and Methods

### Patients

All neurology patients who were hospitalized in the Affiliated Hospital of Guizhou Medical University from September 2015 to December 2017 were retrospectively identified and reviewed in the Medical Image Archiving and Communication System (PACS). Patients were screened according to inclusion criteria: (1) presence of M1 portion of the middle cerebral artery (MCA) stenosis; (2) MRI includes routine sequences, DWI, FLAIR sequence, and magnetic resonance angiography (MRA). Exclusion criteria: (1) peripheral vertigo, encephalitis, hysteria, brain tumors and patients with unknown diagnosis; (2) patients with severe stenosis or occlusion of the posterior circulation; (3) presence of cardiac pacemaker or metal foreign body preventing completion of the MRI examination, or resulting in severe artifacts and non-diagnostic examination; and (4) very early arterial thrombolysis or interventional treatment.

The patients enrolled in the present study were outside of the time window (>6 h) and they were reluctant to receive endovascular treatment. Total acquisition time was <10 min. All patients were admitted to the hospital to improve microcirculation, and were receiving neurotrophic drugs and other conventional concurrent care. All patients charts were reviewed for collection of demographic, clinical, and laboratory data including the following information: age, sex; smoking, alcohol, previous stroke/transient ischemic attack, coronary artery disease, arterial fibrillation; systolic blood pressure, diastolic blood pressure; blood glucose, cholesterol, triglycerides, high density lipoprotein, low density lipoprotein, homocysteine, National Institutes of Health Stroke Scale Score (NIHSS) at admission and discharge. The patients were followed up 3 months after discharge and scored using Modified Rankin Scale (mRS).

### MR Imaging Protocol

MRI examinations were performed using a Philips Achieva X-Series 3.0T superconducting MR scanner and 8-channel SENSE head coil. Axis-position scanning was performed using a single-shot echo planar imaging sequence (SS-EPI) parallel to the anterior commissure-posterior (AC-PC) plane and spanning the entire brain. All patients had cranial MR scans including transverse fast spin echo (FSE) T_2_WI, transverse T_2_ FLAIR, transverse SE T_1_WI, cross-sectional DWI (b = 0, 1,000 s/mm^2^) and 3D time-of-flight (3D TOF) MRA sequences. Specific scanning parameters as follows: (1) FSE T_2_WI: TR = 3,780 ms, TE = 104.5 ms, slice thickness = 6.0 mm, inter-slice gap = 1.8 mm, FOV = 240 × 180 mm, matrix size = 320 × 224, NEX = 1; (2) T_2_ FLAIR: TR = 8,002 ms, TE = 200 ms, TI = 2,000 ms, slice thickness = 6.0 mm, inter-slice gap = 1.8 mm, FOV = 240 × 240 mm, matrix size = 256 × 192, NEX = 1; (3) T_1_WI: TR = 2,459 ms, TE = 27.2 ms, TI = 760 ms, slice thickness = 6.0 mm, inter-slice gap = 6.0 mm, FOV = 240 mm × 180 mm, matrix size = 228 × 192, NEX = 2; (4) DWI: TR = 4,500 ms, TE = 81.7 ms, slice thickness with no inter-slice gap, FOV = 240 × 240 mm, matrix size = 128 × 128, NEX = 2; (5) 3D TOF MRA: TR = 24 ms, TE = 2.9 ms, slice thickness = 1 mm, inter-slice gap = 0.7 mm, FOV = 210 × 185 mm, matrix size = 288 × 192, NEX = 1.

### Image Analysis

**Figure 1:**
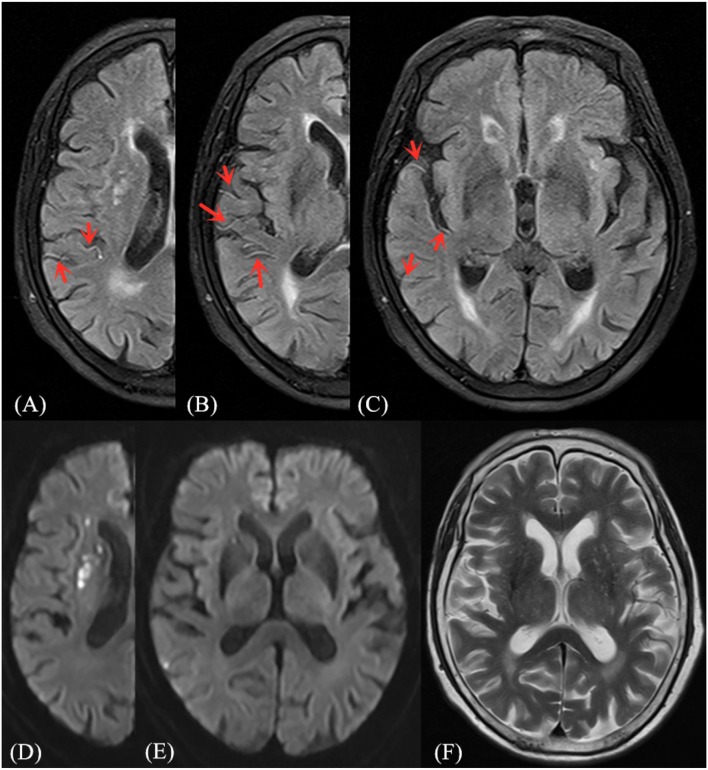
Illustrative case of FVH-DWI mismatch. Magnetic resonance (MR) imaging of a 71-year-old man obtained 1 day after sudden onset of left hemiparesis. Prominent FVH on FLAIR **(A–C)** with small hyperintense lesions in the right MCA territory on admission DWI **(D,E)**, which is more extensive beyond the boundaries of the DWI high signal area, indicating an FVH-DWI mismatch. Prominent FVH presents flow voids on the corresponding T_2_WI image **(F)**.

**Figure 2:**
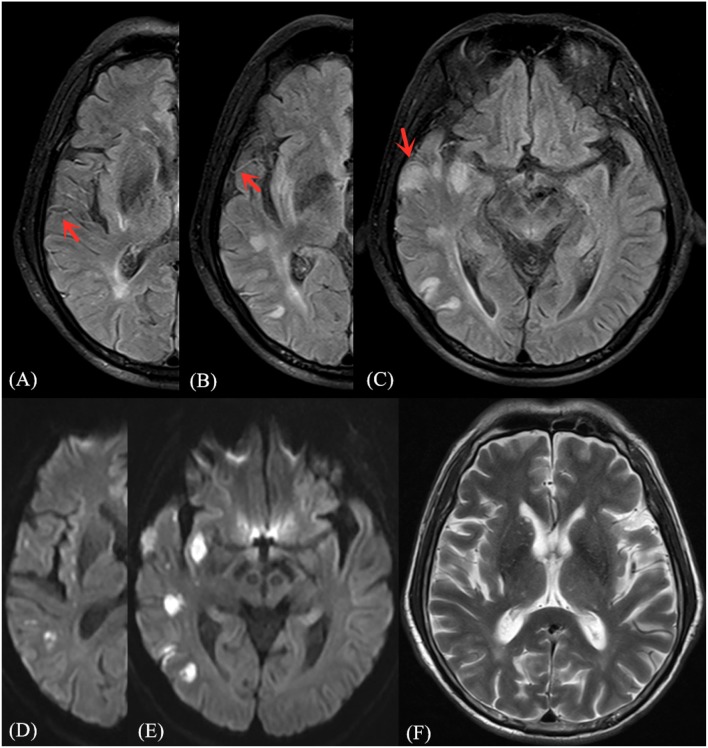
Illustrative case of FVH-DWI match. Magnetic resonance (MR) imaging of a 68-year-old man obtained 2 days after sudden onset of left limb paralysis and speech disorder. Partial prominent FVH on FLAIR **(A–C)** is more extensive within the boundaries of the DWI **(D,E)** high signal area, indicating an FVH-DWI match. Prominent FVH presents flow voids on the corresponding T_2_WI image **(F)**.

FVH was judged by the following standards (13): (1) FVH is defined as a focal, tubular or serpentine hyperintensity on the FLAIR image in the lateral fissure, sulci or near the brain surface; (2) corresponding T2WI image demonstrates flow void; (3) typical signs appear at least on one level. If the above three criteria are satisfied, the FVH sign is positive, otherwise it is considered negative. The FVH score was calculated according to the Olindo et al. (14) method, which was continuously observed from first M1-MCA appearance. The absence of FVH on one slice was rated as 0 point, and when one or more FVHs found on one slice they were rated as 1 point. FVH-DWI mismatch means the FVH signal range exceeds the DWI lesions whereas the hyperintensity area on DWI was excluded when measuring FVH (recorded only FVH outside DWI), and FVH-DWI match refers to the FVH within the hyperintensity area on DWI (recorded FVH only inside DWI) (9) (As shown in Figures 1, 2).

Patients were divided into five groups based on the symptom onset to scan time gap, namely 1 day or less group, 2–4 days group, 5–9 days group, 10–13 days group, more than 14 days group (15). DWI-ASPECTS score quantifies the extent of cerebral infarction in the area of the MCA supply by dividing the area of MCA into 10 at centrum semiovale and basal ganglia area of the cerebral hemisphere. Including the caudate nucleus (C), insula (I), lentiform nucleus (L), internal capsule (IC), anterior cortex of MCA (M1), lateral cortex of MCA (M2), posterior cerebral cortex of MCA (M3), superior cortex of the anterior cerebral cortex of MCA (M4), superior cortex of the lateral cortex of MCA (M5), superior cortex of the posterior cerebral cortex of MCA (M6) (10, 12). Measurement of the degree of vascular stenosis is based on the original images of the 3D-TOF MRA and the reconstructed images. The stenosis rate of MCA is calculated according to the standard of Warfarin-Aspirin for Symptomatic Intracranial Disease study (WASID) (16), that is, the diameter stenosis rate (%) = [1-stenosis diameter/stenosis proximal normal segment diameter] × 100%. Measurement of stenosis rates are averaged three times and the most significant parts are selected for measuring tandem stenosis or multiple stenoses. The stenosis rates are divided into four levels by the above method: (1) mild stenosis, <30%; (2) moderate stenosis, 30–69%; (3) severe stenosis, 70–99%; (4) completely occluded, 100%, no signal on MRA (17).

All MRI images of patients were analyzed and measured by two senior neuroradiologists (GS and BG) with nearly 10 years of working experience without knowing the clinical details. In case of disagreement, a deputy director of neuroradiology participated in interpreting the images and helped to reach consensus. Details on the raw data are reported in Supplementary Material Data Sheet 1.

### Statistical Analysis

All of the analyses were performed with the statistical software packages R (http://www.R-project.org, The R Foundation) and EmpowerStats (http://www.empowerstats.com, X&Y Solutions, Inc., Boston, MA). Continuous variables were presented as mean ± standard deviation (x ±s), and categorical variables expressed as a percentage or frequency. Quantitative data to meet the normal distribution and homogeneity of variance in the two groups were compared using *t*-test or analysis of variance, and the quantitative data do not satisfy the normal distribution at the same time and homogeneity of variance when comparing the two groups using the rank sum test. The group comparison of the categorical variables was compared with the Mann–Whitney and chi-square tests, and the exact probability method of Fisher was adopted when the theoretical frequency was <10. The correlation between DWI-ASPECTS and FVH-DWI mismatch/match was tested using Spearman correlation analysis. Taking the FVH-DWI mismatch/match as the dependent variable, the related independent variables were included in the Logistic regression model, and stepwise regression analysis was used to test meaningful independent prediction indicators. All the test methods were statistically significant with the difference of *P* < 0.05.

## Results

### General Population

According to the inclusion criteria, the number of cases included in this study was 109. There were 66 males and 43 females, with an average age of 64.4 ± 13.2 years. One hundred nine patients were divided into two groups based on areas of FVH and DWI. Forty-three cases were FVH-DWI match, and 43 cases were FVH-DWI mismatch. The main baseline characteristics of clinical data and imaging data of two groups were summarized in Table 1.

**Table 1:** Baseline characteristics in patients with FVH-DWI match and FVH-DWI mismatch.

**Characteristics**	**Total** **(*n* = 109)**	**FVH-DWI match** **(*n* = 43)**	**FVH-DWI mismatch** **(*n* = 66)**	***P*-value**
Age (years)	64.4 ± 13.2	67.4 ± 14.1	62.4 ± 12.2	0.049
Male, *n* (%)	66 (60.6%)	24 (55.8%)	42 (63.6%)	0.414
Systolic BP (mm Hg)	149.1 ± 20.5	149.3 ± 24.8	149.0 ± 17.4	0.953
Diastolic BP (mm Hg)	86.9 ± 12.7	85.1 ± 15.0	88.0 ± 11.0	0.244
Serum glucose (mmol/L)	7.3 ± 3.6	6.8 ± 2.0	7.6 ± 4.3	0.215
Cholesterol (mmol/L)	4.6 ± 1.2	4.5 ± 1.0	4.7 ± 1.3	0.250
Triglycerides (mmol/L)	1.7 ± 0.9	1.5 ± 0.7	1.9 ± 1.0	0.022
HDL (mmol/L)	1.3 ± 0.3	1.3 ± 0.3	1.3 ± 0.3	0.971
LDL (mmol/L)	2.7 ± 1.0	2.6 ± 0.9	2.7 ± 1.1	0.464
Homocysteine (μmol/L)	17.5 ± 6.9	16.3 ± 5.9	18.3 ± 7.4	0.136
Smoking (yes), *n* (%)	45 (41.3%)	17 (39.5%)	28 (42.4%)	0.765
Drinking (yes), *n* (%)	34 (31.2%)	10 (23.3%)	24 (36.4%)	0.149
AF (yes), *n* (%)	25 (22.9%)	15 (34.9%)	10 (15.2%)	0.017
Stroke/TIA (yes), *n* (%)	20 (18.3%)	8 (18.6%)	12 (18.2%)	0.956
CAD (yes), *n* (%)	18 (16.5%)	8 (18.6%)	10 (15.2%)	0.635
Stenosis rates				0.264
<30%, *n* (%)	19 (17.4%)	5 (11.6%)	14 (21.2%)	
30~69%, *n* (%)	7 (6.4%)	3 (7.0%)	4 (6.1%)	
70~99%, *n* (%)	29 (26.6%)	9 (20.9%)	20 (30.3%)	
100%, *n* (%)	54 (49.5%)	26 (60.5%)	28 (42.4%)	
Symptom onset to MR (days)				0.029
<1 days, *n* (%)	9 (8.3%)	3 (7.0%)	6 (9.1%)	
1~4 days, *n* (%)	61 (56.0%)	32 (74.4%)	29 (43.9%)	
5~9 days, *n* (%)	25 (22.9%)	6 (14.0%)	19 (28.8%)	
10~13 days, *n* (%)	5 (4.6%)	1 (2.3%)	4 (6.1%)	
≥14 days, *n* (%)	9 (8.3%)	1 (2.3%)	8 (12.1%)	
FVH scores	3.0 (2.0–5.0)	3.0 (2.0–4.5)	3.0 (2.0–5.0)	0.277
DWI-ASPECTS	6.0 (5.0–8.0)	4.0 (2.5–5.0)	7.0 (6.0–8.0)	<0.001
Initial NIHSS scores	18.0 (16.0–22.0)	17.0(16.0–22.0)	18.0(16.0–22.0)	0.363
Discharge NIHSS scores	16.0 (14.0–20.0)	16.0(13.0–21.0)	16.0 (15.0–19.0)	0.541
mRS score at 3 months	3.0 (3.0–4.0)	4.0 (3.0–6.0)	3.0 (2.0–4.0)	<0.001
3-month mRS ≤ 2, *n* (%)	24 (22.0%)	5 (11.6%)	19 (28.8%)	0.035

*FVH-DWI match, FVH inside DWI-positive area; FVH-DWI mismatch, FVH outside DWI-positive area; HDL, high density lipoprotein; LDL, low density lipoprotein; TIA, transient ischemic attack; AF, arterial fibrillation; CAD, coronary artery disease; DWI-ASPECTS, DWI-Alberta Stroke Program Early CT Score; BP, blood pressure; NIHSS, National Institutes of Health Stroke Scale; and mRS, modified Rankin Scale*.

There was no significant difference between the two groups in gender, systolic blood pressure and diastolic blood pressure, serum glucose, Cholesterol, HDL, LDL, smoking, drinking, CAD, homocysteine, stroke/TIA, stenosis rates, FVH scores, initial NIHSS scores, and discharge NIHSS scores (*P* > 0.05). The following parameters were significantly different between two study groups (*P* < 0.05): age, triglycerides, AF, symptom onset to MRI, DWI-ASPECTS, mRS score at 3 months and the number of 3-month mRS ≤ 2. In the group of FVH-DWI match, the patients were of slightly older age (67.4 ± 14.1) than in the group of FVH-DWI mismatch (62.4 ± 12.2). The levels of triglycerides in former group (1.5 ± 0.7) are lower than the latter (1.9 ± 1.0). FVH-DWI match group had the larger proportion of AF (34.9%) than FVH-DWI mismatch group (15.2%). The incidences of symptom onset to MRI in <1, 1–4, 5–9, 10–13, ≥14 days of FVH-DWI match and FVH-DWI mismatch groups were 7.0% (9.1%), 74.4% (43.9%), 14.0% (28.8%), 2.3% (6.1%), and 2.3% (12.1%), respectively. There was a significant difference among two groups (*P* < 0.029). Compared with the FVH-DWI match group, the DWI-ASPECTS in the FVH-DWI mismatch group was higher (7.0 vs. 4.0, *P* < 0.001), and the clinical prognosis of 3 months after discharge was better (mRS score at 3 months, 3.0 vs. 4.0, *P* < 0.001).

### Univariate Analysis

Taking FVH-DWI match and FVH-DWI mismatch as a dichotomous outcome variable, age, triglycerides, AF, symptom onset to MRI, DWI-ASPECTS, mRS score at 3 months and the number of 3-month mRS ≤ 2 as dependent variables. Univariate analysis of the relationship between the dependent variables and the outcome variables was performed, results as shown in Table 2.

**Table 2:** Univariate analysis—variables associated with odds ratio of FVH-DWI mismatch and FVH-DWI match.

	**Statistics**	**OR**	**95% CI**	***P*-value**
Age (years)	64.4 ± 13.2	1.0	(0.9, 1.0)	0.052
Triglycerides (mmol/L)	1.7 ± 0.9	1.9	(1.1, 3.3)	0.030
AF				
No	84	Reference		
Yes	25	0.3	(0.1, 0.8)	0.019
Symptom onset to MR (days)				
<1 days (%)	9	Reference		
1~4 days (%)	61	0.5	(0.1, 2.0)	0.293
5~9 days (%)	25	1.6	(0.3, 8.3)	0.588
10~13 days (%)	5	2.0	(0.1, 26.7)	0.600
≥14 days (%)	9	4.0	(0.3, 48.7)	0.277
DWI-ASPECTS	5.9 ± 2.3	4.7	(2.5, 8.7)	<0.001
mRS score at 3 months	3.5 ± 1.3	0.4	(0.3, 0.6)	<0.001
3-month mRS				
>2 (%)	85	Reference		
≤ 2 (%)	24	3.1	(1.0, 9.0)	0.041

*OR, odds ratio*.

The age and symptom onset to MRI showed statistically significant difference, however the two dependent variables in univariate analysis were not associated with FVH-DWI mismatch. In addition, the DWI-ASPECTS (OR = 4.7, 95% CI = 2.5–8.7, *P* < 0.001) was strongly related to FVH-DWI mismatch. Compared with FVH-DWI match group, a high level of triglycerides (OR = 1.9, 95% CI = 1.1–3.3, *P* = 0.030) was also associated with a higher risk of FVH-DWI mismatch group. Compared with patients without AF, the risk of FVH-DWI match in patients with AF increased by 70%. Additionally, the number of 3-month mRS ≤ 2 (OR = 3.1, 95% CI = 1.0–9.0, *P* = 0.041) was also relevant to FVH-DWI mismatch.

### Multiple Regression Analysis

**Figure 3:**
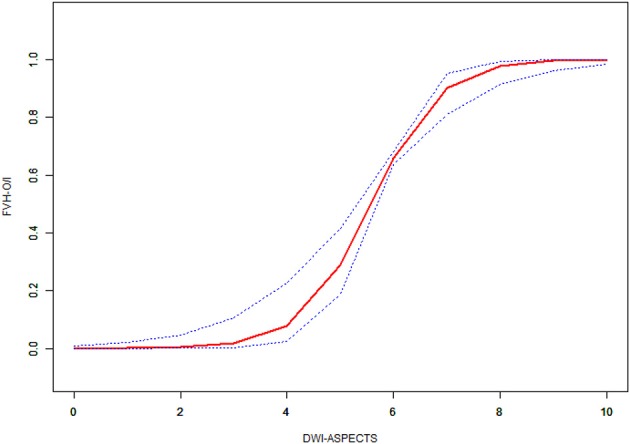
Non-linear association of the difference between DWI-ASPECTS with odds ratio of FVH outside DWI-positive area and FVH inside DWI-positive area by smoothing curve fitting.Outcome: FVH-O/I = FVH-DWI mismatch/FVH-DWI match. Exposure: DWI-ASPECTS.

Multiple regression analysis showed that DWI-ASPECTS (OR = 4.7, 95% CI = 2.5–8.7, *P* < 0.001) was correlated to FVH-DWI mismatch without adjusting for any variables. After adjusting the variables of age and gender, DWI-ASPECTS (OR = 4.7, 95% CI = 2.5–8.6, *P* < 0.001) appeared to represent an independent predictor of FVH-DWI mismatch. Univariate analysis showed that in addition to the correlation between DWI-ASPECTS and odds ratio of FVH-DWI mismatch and FVH-DWI match, homocysteine, AF and the number of 3-month mRS ≤ 2 were also related to FVH-DWI mismatch. After the adjustment of the variables affecting the relationship between DWI-ASPECTS and FVH-DWI mismatch, multiple regression analysis suggested that DWI-ASPECTS (OR = 4.7, 95% CI = 2.5–9.2, *P* < 0.001) remained significantly associated with FVH-DWI mismatch (Table 3). Furthermore, taking FVH-DWI mismatch as the dependent variable, with DWI-ASPECTS as the exposure factor, smooth curve fitting was performed after the controlling of the variables of homocysteine, AF and the number of 3-month mRS ≤ 2 (Figure 3). The curve showed two-stage change and breakpoint. When the DWI-ASPECTS value was < the point of 3, the odds ratio of FVH-DWI mismatch and FVH-DWI match was low; however if the value was more than the point of 8, the odds ratio tended to be high. Between the point of 3–8, the trend of odds ratio was gradually increasing upward.

**Table 3:** Multiple logistic regression analysis of association of DWI-ASPECTS with FVH-DWI mismatch.

**Model**	**DWI-ASPECTS**		
	**OR**	**95% CI**	***P*-value**
Non-adjusted	4.7	(2.5, 8.7)	<0.001
Adjust I	4.7	(2.5, 8.6)	<0.001
Adjust II	4.7	(2.5, 9.2)	<0.001

*Outcome variable: Odds ratio of FVH-DWI mismatch and FVH-DWI match*.

*Exposure variable: DWI-ASPECTS*.

*Non-adjusted model adjust for: none*.

*Adjust I model adjust for: age; gender*.

*Adjust II model adjust for: homocysteine; AF; the number of 3-month mRS ≤ 2*.

## Discussion

Our preliminary study has shown that in patients with MCA stroke: (1) DWI-ASPECTS was independently associated with FVH-DWI mismatch; (2) Patients with FVH-DWI mismatch compared with FVH-DWI match had a better prognosis in 3 months. The above results suggested that FVH-DWI mismatch might be used as an imaging index to assess the prognosis of cerebral infarction caused by unilateral MCA, and that DWI-ASPECTS can differentiate between FVH-DWI mismatch or match independently. Previous studies (5, 7–9, 18) have reported that the presence of FVH distal to a severe vascular stenosis or occlusion may reflect a reversed, slow and static flow in the leptomeningeal circulation in patients with acute ischemic stroke, which was thought to represent an imaging sign of collateral circulation and early ischemia. The investigation by Legrand et al. (7) showed that the volume of DWI lesion in the baseline FVH-DWI mismatch group was smaller than those in FVH-DWI match group in hyperacute cerebral infarction. Our study included patients who were not in the hyperacute stage, and we evaluated infarct size by using ASPECTS instead. Our results showed that DWI-ASPECTS of FVH-DWI mismatch group was higher than that of the match group which is consistent with Legrand et al. (7) study, which indirectly suggests that the prognosis of FVH-DWI mismatch group was better. Therefore, the most important finding of this study is that there is a significant correlation between ASPECTS and FVH-DWI mismatch, which represents an independent predictive metric after adjusting the relevant variables (OR: 4.7; 95% CI: 2.5–9.2; *P* < 0.001).

The innovative part of this study is that DWI-ASPECTS can quantify whether there is a FVH-DWI mismatch by smooth curve fitting. When the DWI-ASPECTS value was < the point of 3, the odds ratio of FVH-DWI mismatch and FVH-DWI match was low; however, if the value was more than the point of 8, the odds ratio of FVH-DWI mismatch and FVH-DWI match tended to be high. Between the point of 3–8, the trend of odds ratio of FVH-DWI mismatch and FVH-DWI match was gradually upward. Penumbra Pivotal Stroke Trial (19) showed that patients with ASPECTS > 7 had better clinical outcomes than ASPECTS ≤ 7, and those with ASPECTS ≤ 4 had poor clinical results. The result of this study is similar to previous related studies (20, 21). Previous studies (7, 8) revealed that FVH-DWI mismatch can predict the prognosis of acute cerebral infarction, however, there is no uniform standard on how to accurately quantify and distinguish between FVH-DWI mismatch and FVH-DWI match. DWI-ASPECTS can quickly and effectively assess the approximate volume of the lesions, and two threshold points for DWI-ASPECTS of 3 and 8 can be used to distinguish whether there is a mismatch in FVH-DWI by our study.

Prior studies confirmed that FVH is common in the early stages of acute cerebral infarction, and that with passage of time, the incidence of FVH gradually decreased (22–24). Maeda et al. (15) included 40 cases of acute and subacute MCA patients with cerebral infarction which were followed up for different time periods. They found that the positive rate of FVH in <24 h, 1–4 and 5–9 days was about 100, 40, and 18%, respectively. Our result are similar to previous study (15) apart from the numbers of acute cerebral infarction, however regression analysis showed that the incidence of FVH was not correlated in match or mismatch group. All the patients with FVH positive sign were included in this study. Therefore, the FVH sign in the two groups did not change with time. Previous studies have confirmed that FVH-DWI mismatch can be used as an indirect imaging marker to better reflect the hyperacute ischemic penumbra (5, 7, 9). At <1 day, most patients were in the hyperacute or acute early stage, and the proportion of patients in the FVH-DWI mismatch group was slightly higher than match group due to the presence of more ischemic penumbra. At 1–4 days, the collateral vessels in the infarct area were established, and FVH reflected the formation of collaterals. Therefore, the proportion of patients in the two groups should be similar in theory, however the results showed that the FVH-DWI mismatch group was significantly < the FVH-DWI match group. It was speculated that the ischemic penumbra plays a leading role (22). The ischemic penumbra gradually transformed into the infarct core region over time, thus resulting in a significant reduction in the proportion of patients in the FVH-DWI mismatch group. After 5–9, 10–13, and ≥14 days, the proportion of patients in the FVH-DWI mismatch group was > that in the FVH-DWI match group, presumably due to the stable collateral vessel formation and the role of poor ischemic penumbra in these stages (9, 23). Therefore, FVH-DWI mismatch was mainly dominated by the collateral circulation, which more reflected the better stable collateral circulation of ischemic stroke.

Similarly, past studies suggested that the incidence of FVH is positively correlated with the degree of arterial stenosis, and therefore the incidence of FVH in patients with severe stenosis or occlusion was significantly higher (25). One of the reasons for this phenomenon is that we included patients with lesions of MCA and fewer patients with acute cerebral infarction in this retrospective study and that FVH-DWI mismatch or match groups were all based on positive FVH signs. Therefore, the incidence of FVH is not time-dependent. In order to further confirm the incidence of FVH after the acute phase in this study was not associated with the onset and the degree of vascular stenosis, we quantified FVH scores (14) for FVH-DWI mismatch and match group and found no significant difference. These findings suggest that DWI-FVH mismatch can persist after the acute phase, and we speculate that the reason is that there may be a better collateral circulation. Prior similar studies revealed that distal FVH indicate a good collateral flow, smaller infarct volume, larger ischemic penumbra and decreased neurological deficit associated with the lesion (26). However, other investigators believed that the distal FVH is not related to the severity of stroke, or that FVH indicated a lack of collateral circulation (27), which was associated with larger volume of infarction, severe neurological impairment and early deterioration of neurological function (28, 29), and increased the risk of intracranial hemorrhage (22). These different conclusions may be related to the various patient populations studied, inclusion criteria, and different FVH assessment methods. In this study, we found no significant difference between NIHSS admission and NIHSS discharge in FVH-DWI mismatch group and FVH-DWI match group. However the number of patients with mRS ≤ 2 in the FVH-DWI mismatch group was > in the FVH-DWI match group and the ASPECTS scores were also higher. We surmised that the NIHSS score might reflect the severity of stroke, however there was a deviation in patients with coma or stroke recovery, whereas most of the patients included in this study were subacute and chronic. However, mRS is used to assess the prognosis of stroke patients and the level of functional disability in patients in rehabilitation (30), the findings of Legrand et al. (7, 9) also support this viewpoint. They found that the clinical outcome of mRS in FVH-DWI mismatch group is better than that in FVH-DWI mismatch group at 90 days after discharge, which reflect the formation of distal collateral circulation (9). An additional finding in this study was baseline data showing that patients with AF are more likely to develop in FVH-DWI match. Compared with patients without AF, the risk of FVH-DWI match in patients with AF increased by 70%, we speculate that the reason for this may be the insufficiency of collateral circulation around the infarct area or the smaller branch embolism, which is likely to cause a large area infarction, resulting in poor prognosis. Su (31) studied 144 cases of cardiogenic cerebral infarction and found that AF can further reduce cerebral perfusion blood flow and accelerate the progression of infarct, so the prognosis of patients is poor. The above studies confirmed that FVH-DWI mismatch with atrial fibrillation is a risk factor for poor prognosis. In addition, triglycerides levels were found to be higher in the FVH-DWI mismatch group than in the FVH-DWI match group. Univariate results showed that for every 1 μmol/L increase of triglycerides, the risk of FVH-DWI mismatch was increased by 90% compared with that of FVH-DWI match. The possible reason is that risk factor of triglycerides affect the formation of neovascularization and the opening of collateral circulation (32).

This study has the following limitations. First, this study is based on a single center retrospective study and the results need to be confirmed by multicenter, large sample studies. Second, the majority of MRI examinations in both groups were performed in patients with subacute or chronic symptoms and lacked acute or hyperacute MR imaging and analysis, and subjective judgment in the analysis of imaging signs may be present. Third, we only included intracranial MRA acquisition and failed to obtain extracranial carotid artery MRA, and FVH-DWI mismatch in some patients may be caused by the extracranial carotid artery pathology, which may result in bias. Additionally, it is difficult to determine the degree of vascular stenosis in cases of bilateral or multiple vessel disease. Last but not least, DWI-ASPECTS are a semi-quantitative scoring method to indirectly determine the infarct size. The main disadvantage of this technique is that smaller ischemic lesions are also involved in scoring, and this score does not completely reflect the true infarct size.

## Conclusions

FVH-DWI mismatch represents the peripheral blood supply to the infarct, which may be helpful in clinical assessment of infarct size. The DWI-ASPECTS score was an independent predictor of FVH-DWI mismatch. FVH-DWI mismatch did not predict the prognostic value when DWI-ASPECTS ≤ 3, but FVH-DWI mismatch predicts the highest prognostic value when DWI-ASPECTS ≥ 8. DWI-ASPECTS can roughly determine whether there is a FVH-DWI mismatch in order to select optimal clinical treatment and accurately assess prognosis.

## Data Availability Statement

The raw data supporting the conclusions of this manuscript will be made available by the authors, without undue reservation, to any qualified researcher.

## Ethics Statement

This studies involving human participants were reviewed and approved by Ethics Committee of the Affiliated Hospital of Guizhou Medical University. The patients/participants provided their written informed consent to participate in this study.

## Author Contributions

LS and BG: literature search, figures, study design, data collection, data analysis, data interpretation, and writing. CL: study design, data collection, and data analysis. GS, JW, and WW: data analysis. TG: study design. XQ and MW: data analysis and grammar. AL: manuscript review, revision and proofreading.

### Conflict of Interest

The authors declare that the research was conducted in the absence of any commercial or financial relationships that could be construed as a potential conflict of interest.
