# Supplementary material for: Application of FLAIR Vascular Hyperintensity-DWI Mismatch in Ischemic Stroke Depending on Semi-Quantitative DWI-Alberta Stroke Program Early CT Score
Source: Front Neurol. 2019 Sep 26;10:994. doi: 10.3389/fneur.2019.00994 (PMC6776088; doi:10.3389/fneur.2019.00994)
Supplement: Supplementary file 1 [file Data_Sheet_1.pdf]

Raw data

Patient	Symptom onset	Age	Sex	Smoking	Drinking	Systolic BP	Diastolic BP
1	1~4	70	male	No	No	150	98
2	1~4	68	female	No	No	175	94
3	1~4	69	female	No	No	169	91
4	1~4	79	male	Yes	No	184	112
5	> 14~9	57	male	Yes	Yes	154	86
6	> 14	70	male	No	No	140	78
7	> 14~9	67	male	Yes	Yes	150	76
8	> 14	71	female	No	No	137	82
9	1~4	85	male	No	No	134	73
10	< 1	66	female	No	No	138	71
11	> 14~9	50	male	Yes	Yes	164	94
12	> 14	71	male	No	No	128	84
13	1~4	71	female	No	No	153	100
14	> 14~9	48	female	No	No	178	90
15	> 14	50	male	Yes	Yes	162	93
16	1~4	59	male	Yes	Yes	180	99
17	1~4	66	male	Yes	Yes	130	77
18	> 14	79	female	No	No	140	100
19	1~4	63	female	No	No	176	98
20	1~4	44	male	Yes	Yes	107	71
21	> 14	46	male	Yes	Yes	143	83
22	1~4	70	female	No	No	125	74
23	> 14	74	male	No	No	142	86
24	1~4	81	female	No	No	177	96
25	1~4	67	female	No	No	142	90
26	1~4	52	male	No	No	170	115
27	1~4	75	male	No	Yes	155	80
28	1~4	60	male	Yes	No	160	102
29	> 14~9	65	male	Yes	No	132	78
30	1~4	41	female	Yes	Yes	87	46
31	> 14~9	53	male	No	No	148	102
32	< 1	69	male	No	No	180	94
33	1~4	59	male	Yes	Yes	161	97
34	1~4	75	female	No	No	160	101
35	< 1	70	male	Yes	Yes	164	98
36	1~4	68	male	No	No	137	86
37	1~4	59	male	Yes	Yes	186	117
38	1~4	69	female	No	No	156	82
39	> 14~9	68	female	No	No	150	78
40	1~4	61	male	Yes	Yes	170	112
41	1~4	50	male	Yes	Yes	136	96
42	> 14~9	51	male	Yes	Yes	155	75
43	< 1	69	male	Yes	No	153	97
44	1~4	75	female	No	No	150	80
45	1~4	97	female	No	No	158	76
46	> 14~9	37	male	Yes	Yes	148	94
47	1~4	84	male	Yes	No	120	80
48	1~4	74	male	Yes	Yes	158	92
49	1~4	67	male	Yes	No	170	110
50	1~4	49	female	No	No	125	80
51	1~4	69	male	No	No	114	77
52	1~4	76	male	No	No	170	95
53	> 14	53	female	No	Yes	184	104
54	1~4	69	male	No	No	160	84

55	1~4	48	female	No	No	134	95
56	1~4	67	male	Yes	Yes	153	82
57	1~4	73	male	No	Yes	187	103
58	< 1	42	male	Yes	Yes	165	114
59	1~4	86	female	No	No	155	79
60	1~4	63	female	No	No	140	86
61	1~4	89	female	No	No	150	70
62	1~4	85	female	No	No	149	109
63	< 1	68	female	No	No	147	63
64	> 14~9	50	female	No	No	134	82
65	> 14~9	55	male	Yes	No	187	110
66	> 14~9	63	female	No	No	163	86
67	> 14~9	48	female	No	No	120	80
68	1~4	67	male	Yes	Yes	114	80
69	1~4	65	male	Yes	Yes	154	96
70	> 14~9	60	male	Yes	No	156	92
71	> 14~9	76	male	No	No	160	90
72	10~13	62	male	Yes	Yes	130	80
73	> 14~9	47	male	Yes	No	114	78
74	1~4	34	male	Yes	Yes	137	80
75	1~4	83	female	No	No	145	80
76	1~4	63	male	Yes	No	180	90
77	1~4	61	male	Yes	No	127	73
78	< 1	69	female	No	No	180	75
79	1~4	58	male	Yes	Yes	150	90
80	> 14~9	62	male	No	Yes	158	82
81	1~4	56	male	Yes	Yes	136	90
82	1~4	76	female	No	No	160	95
83	1~4	83	male	Yes	No	130	78
84	1~4	47	male	Yes	Yes	183	102
85	1~4	77	female	No	No	124	63
86	> 14~9	40	male	No	No	134	78
87	10~13	46	female	No	No	119	86
88	1~4	80	male	Yes	No	176	90
89	1~4	50	male	No	No	131	69
90	> 14~9	75	male	Yes	Yes	176	82
91	1~4	71	male	Yes	No	132	78
92	10~13	66	female	No	No	120	70
93	> 14~9	85	female	No	No	133	94
94	10~13	59	female	No	No	145	85
95	10~13	48	male	Yes	Yes	138	96
96	1~4	88	female	No	No	170	80
97	1~4	48	male	No	No	120	80
98	> 14	77	female	No	No	155	89
99	1~4	39	male	Yes	No	120	75
100	> 14~9	81	female	No	No	150	90
101	< 1	45	male	No	No	135	84
102	1~4	80	male	No	No	121	60
103	1~4	58	female	No	No	150	90
104	> 14~9	55	male	Yes	Yes	156	78
105	1~4	72	female	No	No	150	90
106	< 1	72	male	Yes	Yes	173	101
107	> 14~9	52	male	No	No	138	80
108	> 14~9	64	female	No	No	168	88
109	> 14~9	77	female	No	No	126	60

Serum glucose	AF	Stroke/TIA	CAD	Cholesterol	Triglycerides	HDL	LDL
5.79	No	Yes	No	3.43	1.41	1.14	1.65
6.46	No	No	No	5.27	1.84	1.13	3.3
6.05	Yes	No	No	4.65	1.1	1.25	2.9
9.88	Yes	Yes	Yes	5.22	0.94	1.83	2.96
4.78	No	No	No	6.58	1.92	1.1	4.61
4.56	No	No	No	4.76	3.08	0.95	2.41
4.78	No	No	No	4.74	2.24	1.32	2.4
5.69	Yes	No	No	5.93	1.38	1.95	3.35
4.9	Yes	Yes	Yes	4.3	0.73	1.42	2.55
5.21	No	No	No	4.21	0.92	1.24	2.37
5.99	No	No	No	3.59	2.24	0.8	1.77
14.45	No	No	Yes	2.88	1.72	1.31	0.79
5.29	No	No	No	5.95	1.81	1.68	3.45
6.09	No	No	No	4.84	1.83	1.28	2.73
3.63	No	No	No	3.38	1.16	0.98	1.87
4.38	No	No	Yes	4.76	1.21	1.32	3.12
5	No	No	No	4.36	2.18	1.45	1.92
8.98	Yes	Yes	Yes	3.87	0.64	1.77	1.81
6.15	No	Yes	No	3.96	1.3	1.08	2.29
4.61	No	No	No	4.92	0.67	1.71	2.91
19.99	No	No	No	4.58	1.79	1.01	2.76
7.57	Yes	No	No	4.04	1.13	1.03	2.5
5.98	No	No	No	4.57	1.65	1.27	2.55
11.62	No	No	Yes	4.54	1.95	1.26	2.39
11.07	No	No	Yes	3.95	1.33	1.33	2.02
5.06	No	Yes	No	4.7	5.24	1.26	1.06
8.71	No	No	No	4.15	1.47	1.41	2.07
6.18	No	No	No	5.77	1.17	1.39	3.85
4.64	No	No	No	4.93	1.24	1.22	3.15
6.2	No	No	No	4.93	1	1.39	3.09
7.07	No	No	No	3.19	3.09	0.91	0.88
4.27	Yes	No	No	2.61	1.1	0.91	1.2
7.94	Yes	No	Yes	3.73	2.69	0.83	1.68
6.48	No	No	Yes	6.63	1.9	1.7	4.07
13.57	No	No	Yes	6.83	1.78	1.57	4.45
7.74	No	No	No	4.26	1.65	0.8	2.71
4.49	Yes	No	No	2.32	1.94	0.61	0.83
6.16	No	No	No	4.63	0.72	1.67	2.63
28.37	No	Yes	No	6.33	5.18	1.36	2.62
7.31	No	No	No	4.22	1.85	1.58	1.8
5.03	No	No	No	7.59	5.43	1.58	3.54
15.61	No	No	No	5.01	1.75	1.41	3.39
7.16	No	No	No	4.69	1.29	1.35	2.75
6.89	No	Yes	No	4.61	1.82	1.04	2.74
8.23	No	No	Yes	4.38	0.63	2.15	1.94
6.08	No	No	No	3.19	2.28	0.75	1.4
5.44	Yes	No	Yes	2.62	1.18	0.75	1.33
7.02	No	No	No	4.18	1.63	1	2.44
13.8	No	No	Yes	5.45	3.44	1.32	2.57
6.05	Yes	No	No	4.89	0.83	1.29	3.55
5.28	Yes	No	No	3.58	0.58	1.39	2
5.07	No	No	No	2.84	1.31	0.82	2.04
11.77	No	No	No	4.62	4.13	1.29	2.2
6.53	No	No	No	5.63	1.58	1.12	4.47

6.04	Yes	No	No	4.07	1.03	1.13	3.11
3.83	No	No	No	4.08	1.25	0.97	3.37
7.92	No	No	No	3.89	0.91	1.8	2.37
4.53	No	No	No	2.18	2.11	1.21	3.5
7.9	No	No	No	5.63	1	1.79	3.69
5.82	No	Yes	No	4.64	1.14	1.83	2.56
6.55	Yes	No	No	4.17	0.82	1.56	2.2
12.02	Yes	Yes	Yes	4.71	2.21	1.07	3.19
11.5	No	No	No	4.36	1.64	1.35	2.71
4.96	No	No	No	5.26	2.21	1.1	3.16
6.67	No	No	No	6.74	2.03	1.28	4.54
5.32	No	No	No	6.32	2.85	1.58	3.44
4.38	No	No	No	3.25	1.81	0.82	1.61
9.94	No	No	No	3.36	1.7	1.22	1.37
4.35	Yes	No	No	6.4	1.22	1.55	4.3
6.21	No	No	No	5.29	1.63	1.34	3.21
7.32	No	No	No	5.99	1.86	1.29	3.85
5.21	No	No	No	4.61	1.93	1.28	2.45
5.2	No	No	No	3.17	1.17	0.99	1.65
7.22	Yes	No	No	5.31	1.14	0.78	4.01
4.82	No	No	No	4.18	1.77	1.06	2.32
6.78	No	No	No	4.74	2.05	1.39	2.42
8.67	No	No	No	4.77	1.62	1.18	2.85
7.87	No	No	No	4.76	1.78	1.22	2.73
5.76	No	Yes	No	6.97	2.35	1.4	5.5
9.27	No	Yes	No	3.98	0.56	1.57	2.16
7.58	No	No	No	4.98	2.35	0.97	2.94
6.59	Yes	No	No	6.77	2.01	1.51	4.35
5.52	No	No	No	3.33	0.92	0.76	2.15
6.55	No	No	No	5.72	2.22	1.19	3.52
6.84	No	Yes	No	5.65	0.71	1.39	3.94
4.7	No	No	No	3.98	1.14	0.89	2.52
4.34	No	No	No	3.82	1.17	1.13	2.16
7.4	No	No	No	5.13	2.11	1.08	3.09
13.68	No	No	No	2.73	1.45	0.86	1.21
6.08	No	No	No	3.6	0.74	1.54	1.72
10.63	Yes	No	No	2.42	0.83	0.86	1.18
4.09	Yes	Yes	No	2.3	0.87	0.95	0.95
7.74	Yes	Yes	Yes	4.38	1.85	1.11	2.43
5.19	No	No	No	3.99	1.59	1.13	2.14
7.5	No	No	No	5.69	1.76	1.22	3.67
7.18	No	No	No	6.6	1.46	1.51	4.43
4.11	No	Yes	No	4.51	1.77	1.11	2.6
5.37	No	No	Yes	5.09	2.12	1.56	3.05
5.53	No	No	No	4.71	2.2	1.05	3.26
5.6	No	Yes	No	6.32	2.17	1.03	3.3
6.79	No	No	No	3.9	2.32	1.03	1.82
6.4	Yes	No	Yes	3.3	0.73	1.76	1.21
9.58	No	Yes	No	6.35	2.62	1.37	3.79
5.16	No	No	No	3.92	1.91	1.03	2.02
6.04	Yes	Yes	Yes	4.93	1.91	1.12	1.13
11.8	Yes	No	No	3.89	0.88	1.19	2.3
5.41	No	Yes	No	5.09	1.79	0.78	3.5
16.64	No	No	No	8.96	4.61	1.58	5.28
5.81	Yes	No	No	4.38	0.9	1.67	2.3

Homocysteine	Stenosis rates	FVH-DWI	FVH scores	DWI-ASPECTS	mRS	Initial NIHSS
19.98	70～99%	mismatch	3	7	3	7
20.75	30 ～69%	match	1	4	6	16
10.74	30 ～69%	mismatch	4	5	4	18
21.85	100%	match	1	0	6	26
42.59	100%	mismatch	4	9	1	17
16.23	100%	mismatch	3	7	4	19
23.32	100%	match	2	5	3	22
17.21	0 ～29%	mismatch	2	9	3	21
18.81	100%	match	3	5	2	26
15.12	100%	mismatch	6	7	2	16
18.66	100%	mismatch	5	8	4	13
14.4	100%	mismatch	4	8	3	17
19.05	0 ～29%	mismatch	1	9	4	22
8.49	70～99%	mismatch	6	7	2	22
11.23	70～99%	mismatch	2	9	2	12
15.21	100%	mismatch	4	4	3	22
17.25	0 ～29%	mismatch	2	10	1	17
10.36	100%	mismatch	4	6	4	18
17.36	100%	match	2	1	6	26
27.61	70～99%	match	1	5	3	10
9.81	100%	mismatch	5	7	5	16
17.42	70～99%	mismatch	3	8	3	18
18.03	100%	mismatch	6	9	2	19
11.12	100%	match	3	2	6	16
9.26	70～99%	mismatch	2	8	3	17
15.21	0 ～29%	mismatch	1	9	4	18
26.87	30 ～69%	mismatch	3	8	3	12
28.4	100%	mismatch	3	8	3	14
30.32	100%	mismatch	4	6	4	12
9.21	70～99%	match	2	2	6	22
17.75	70～99%	mismatch	5	7	3	18
20.33	100%	match	2	0	6	21
15.07	100%	match	3	3	6	16
17.04	70～99%	mismatch	2	8	4	22
14.36	100%	mismatch	4	7	3	20
14.02	70～99%	match	3	6	5	12
12.23	100%	match	3	0	6	30
7.24	100%	match	3	5	4	20
14.78	70～99%	mismatch	2	7	3	16
13.91	100%	match	5	4	5	17
19.82	30 ～69%	mismatch	5	7	2	17
17.23	70～99%	mismatch	1	5	3	20
13.38	100%	match	5	0	6	16
17.37	70～99%	match	3	5	5	16
14.56	100%	match	2	1	4	17
15.96	70～99%	mismatch	3	6	3	23
19.02	100%	mismatch	5	7	2	17
12.95	0 ～29%	mismatch	2	8	3	22
21.9	0 ～29%	mismatch	2	9	3	23
10.06	30 ～69%	match	3	3	6	25
15.03	100%	match	5	1	6	25
23.8	70～99%	match	3	6	4	26
8.1	0 ～29%	match	2	8	3	16
15.08	100%	match	3	5	5	17

19.11	100%	mismatch	5	5	3	23
8.02	100%	match	5	4	5	15
10.11	0 ~29%	match	1	6	3	11
41.84	100%	mismatch	3	6	4	22
16.6	0 ~29%	mismatch	2	9	2	9
21.44	100%	mismatch	8	5	2	21
9.02	0 ~29%	match	2	5	5	16
28.53	100%	match	2	4	4	27
14.12	70 ~ 99%	mismatch	5	8	2	11
14.73	0 ~29%	mismatch	4	8	1	17
17.26	70 ~ 99%	mismatch	2	7	3	15
14.11	70 ~ 99%	match	8	6	3	22
12.92	100%	mismatch	2	7	4	22
11.65	0 ~29%	mismatch	4	9	4	20
18.19	70 ~ 99%	mismatch	1	6	3	25
20.17	100%	match	5	5	1	18
22.01	0 ~29%	mismatch	2	4	3	22
17.13	70 ~ 99%	mismatch	7	8	2	23
13.08	100%	match	7	4	1	22
18.03	100%	match	4	4	3	20
18.21	100%	mismatch	3	6	4	22
17.19	70 ~ 99%	mismatch	4	6	5	22
16.78	0 ~29%	match	2	5	3	18
16.78	100%	match	5	6	2	17
11.89	100%	mismatch	7	9	2	13
9.75	0 ~29%	mismatch	8	8	3	22
18.5	30 ~69%	mismatch	2	6	4	16
21.43	100%	match	2	5	3	15
7.38	0 ~29%	match	2	5	5	21
47.13	100%	mismatch	4	8	4	16
17.14	100%	match	4	0	5	17
12.78	100%	mismatch	3	7	2	23
20.6	70 ~ 99%	match	5	5	3	17
31.74	70 ~ 99%	match	3	4	4	22
22.01	100%	mismatch	5	7	3	23
26.79	0 ~29%	mismatch	1	9	4	15
21.98	100%	match	4	5	4	15
14.24	70 ~ 99%	mismatch	3	7	2	21
14.31	100%	mismatch	3	5	3	19
14.77	70 ~ 99%	mismatch	2	9	4	17
12.57	100%	mismatch	4	7	3	13
19.39	70 ~ 99%	mismatch	3	8	2	12
16.65	100%	mismatch	3	6	3	16
30.24	100%	mismatch	5	6	4	18
17.67	100%	match	6	4	2	8
10.82	100%	match	5	2	4	21
21.98	70 ~ 99%	mismatch	5	9	2	22
18.74	30 ~69%	match	1	5	4	8
19.18	100%	mismatch	4	6	3	16
22.27	100%	mismatch	7	5	4	12
8.98	100%	match	4	5	5	20
11.97	0 ~29%	mismatch	6	7	2	22
17.3	70 ~ 99%	mismatch	2	6	4	18
16.73	0 ~29%	mismatch	2	6	4	7
17.96	70 ~ 99%	match	3	4	6	16

Discharge NIHSS
1
15
16
28
15
18
24
20
23
15
14
18
20
22
16
20
15
16
23
12
15
18
16
14
14
13
12
14
19
20
14
19
12
19
18
9
25
18
15
15
15
16
12
15
16
22
16
22
22
24
23
22
15
15

21
13
12
19
4
18
15
26
10
15
15
19
22
18
20
15
21
22
22
20
19
20
14
9
12
19
12
12
18
15
16
22
16
22
22
14
13
18
16
15
13
12
15
16
10
20
16
6
15
16
18
20
13
9
12
